# *Costunolide*‐Mediated Modulation of the P2X7–NLRP3 Complex Suppresses Oligodendrocyte Precursor Cell Pyroptosis and Rescues Perinatal White Matter Injury

**DOI:** 10.1002/cns.71263

**Published:** 2026-11-09

**Authors:** Feng Zhang, Qian Wang, Ting Li, Xianghong Li, Mengya Sun, Liangliang Li, Miao Qin, Shiyi Ma, Junfang Sun, Wenyu Yu, Yan Liu, Hong Jiang

**Affiliations:** ^1^ Department of Pediatrics, Pediatric Research Institute, Children's Medical Center The Affiliated Hospital of Qingdao University Qingdao Shandong China

**Keywords:** *Costunolide*, NLRP3 inflammasome, oligodendrocyte precursor cells, P2X7 receptor, perinatal white matter injury, pyroptosis

## Abstract

**Background:**

Perinatal white matter injury (WMI) causes neurodevelopmental disability, driven by oligodendrocyte precursor cell (OPC) pyroptosis. We aimed to identify the molecular mechanism of NLRP3 inflammasome assembly in OPCs and evaluate *Costunolide* (Cos) as a targeted modulator of the P2X7–NLRP3 complex.

**Methods:**

We integrated molecular dynamics simulations, surface plasmon resonance, primary OPC cultures, single‐cell RNA sequencing, and a custom CRISPR/Cas9‐generated *P2rx7* knockout rat model to assess Cos efficacy.

**Results:**

Cos associated with the extracellular domain of P2X7 (*K*_D_ = 3.08 μM, *Homo sapiens* P2X7), modulating the P2X7–NLRP3 complex, thereby restricting calcium influx and preventing Caspase‐1 activation and GSDMD‐mediated membrane pore formation in vitro. Single‐cell transcriptomic analysis identified an injury‐induced *P2rx7* ^+^/*Nlrp3* ^+^ OPC subpopulation, revealing a previously unrecognized cell‐intrinsic susceptibility to neuroinflammatory injury. Both genetic deletion of *P2rx7* and pharmacological treatment with Cos markedly suppressed pyroptotic signaling in vivo, resulting in sustained restoration of myelin integrity and enhanced oligodendrocyte maturation from the acute (P7) to the developmental recovery (P14) stage.

**Conclusions:**

Our findings suggest that P2X7 may facilitate NLRP3 inflammasome assembly in developing OPCs. Consequently, modulation of this complex by Cos highlights a potential neurotherapeutic strategy for perinatal WMI.

## Graphical abstract

Perinatal white matter injury compromises developmental myelination through P2X7–NLRP3–driven pyroptosis in oligodendrocyte precursor cells. *Costunolide* (Cos) binds the P2X7 extracellular domain and suppresses NLRP3‐dependent pyroptosis, partially restoring myelination in a rat model. This intervention mitigates inflammatory damage and preserves myelin integrity in vivo.

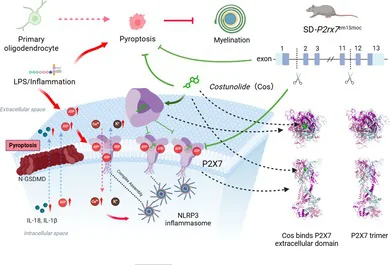

## 1 Introduction

Perinatal white matter injury (WMI) is a leading cause of lifelong neurodevelopmental disability in preterm infants, yet effective early interventions remain unavailable [1]. Oligodendrocyte precursor cells (OPCs) are especially vulnerable in the injured developing brain; their depletion and impaired maturation result in dysmyelination and disrupted white matter integrity [1, 2, 3].

Recently, our understanding of cell death in the oligodendrocyte lineage has expanded to include pyroptosis. In contexts ranging from Alzheimer's disease [4] to hypoxic–ischemic injury [5, 6], both mature oligodendrocytes and OPCs have been shown to activate the NOD‐like receptor family pyrin domain‐containing 3 (NLRP3) inflammasome. Nevertheless, the molecular switch that initiates NLRP3 assembly in developing OPCs remains poorly defined. The purinergic receptor P2X7, an extracellular ATP sensor, is a critical mediator of neuroinflammation [7]. Similarly, in developing OPCs, its activation drives intracellular calcium (Ca^2+^) overload and macropore formation, leading to acute cytotoxicity [8]. Despite the P2X7/NLRP3 axis emerging as a central inflammatory conduit in diverse pathologies—such as cardiac hypertrophy [9] and enthesis regeneration [10]—neuroimmunological studies typically frame P2X7 strictly as a passive ion channel. In this conventional model, the receptor is presumed to induce inflammasome activation only indirectly, via Ca^2+^ influx or potassium (K^+^) efflux. This restricted view has arguably constrained the rational design of targeted structural therapies.

To identify new therapeutic avenues, we conducted a phenotype‐based screen and found that *Costunolide* (Cos), a sesquiterpene lactone, robustly protects OPCs during inflammatory challenge. Although Cos has been reported to act via diffuse intracellular signaling networks [11, 12, 13], our biophysical and structural analyses identified a potential binding site on the extracellular domain of P2X7. We hypothesized that P2X7 may facilitate NLRP3 inflammasome assembly at the plasma membrane. Using *P2rx7* knockout rats and single‐cell transcriptomics, we tested whether disrupting this receptor‐anchored complex—pharmacologically or genetically—halts OPC pyroptosis and perinatal WMI. Our findings support a “receptor‐anchored inflammasome assembly” model, in which Cos both blocks ion flux and modulates the P2X7–NLRP3 complex, offering a translatable strategy.

## 2 Methods

### 2.1 Molecular Docking and Molecular Dynamics Simulations

The crystal structures of *Homo sapiens* (PDB ID: 9E3N [14]) and *Rattus norvegicus* (PDB ID: 6U9W [15]) P2X7 receptors in the ATP‐bound open state were retrieved from the Protein Data Bank. Both structures were preprocessed with PyMOL and PDBFixer to remove water molecules and non‐functional heteroatoms, repair missing residues, and adjust protonation states to pH 7.4, while preserving homotrimeric architecture. The 3D structure of *Costunolide* (Cos; PubChem CID: 5281437) was energy‐minimized with the MMFF94 force field. Hydrogen atoms and Gasteiger charges were assigned using OpenBabel 2.3.2, and the ligand was converted to PDBQT format. Molecular docking was performed with AutoDock Vina, centering the grid on the extracellular ATP‐binding domain. The top‐ranked poses for both human and rat P2X7–Cos complexes were used as initial coordinates for MD simulations.

All molecular dynamics (MD) simulations [16] were run in GROMACS (2020.6/2021.3) with GPU acceleration and the CHARMM36‐jul2022 force field. Trajectories were processed using GROMACS utilities and DuIvyTools [17] to calculate RMSD, RMSF, radius of gyration, hydrogen bond occupancy, and SASA. Binding free energies were computed by MMPBSA (gmx_MMPBSA v1.6.4), decomposing energies into ΔVDWAALS, ΔEEL, ΔGSOLV, and ΔTOTAL. Free energy landscapes were generated from 2D histograms of RMSD and Rg (32 × 32 grid) using custom Python scripts. Trajectory videos were rendered with PyMOL and ffmpeg (30 fps, 720p) to show longitudinal and transverse views of ligand binding.

### 2.2 Surface Plasmon Resonance (SPR) Assay

Binding affinities were measured at 25°C on a BIAcore 1 K instrument (Cytiva) using CM5 sensor chips [18]. Recombinant human P2RX7 protein (N‐terminal hFc tag, 59.2 kDa, > 95% purity; Sangon Biotech) was immobilized on flow channels 2 and 4 after EDC/NHS activation; channels 1 and 3 served as reference surfaces. A solvent correction curve was established using running buffer (1× PBS‐P+, pH 7.4, 5% DMSO). *Costunolide* (Cos; TargetMol) was serially diluted (0.3125–10 μM) in running buffer and injected at 30 μL/min for 150 s. Regeneration was performed with 10 mM glycine‐HCl (pH 2.0) for 5 min. Sensorgrams were double‐referenced and globally fitted to a 1:1 Langmuir binding model using Biacore Insight software to determine association rate (K_a_), dissociation rate (K_d_), and equilibrium dissociation constant (K_D_).

### 2.3 Single‐Cell RNA Sequencing Data Processing and OPC Subcluster Analysis

Publicly available scRNA‐seq data (GEO: GSE225580 [19]) from sham and hypoxia‐ischemia‐injured neonatal mouse brains were processed with Scanpy in Python 3.10. After quality control, counts were normalized to 10,000 reads per cell and log‐transformed. Highly variable genes were identified (min_mean = 0.0125, max_mean = 3, min_disp = 0.5) and scaled (max_value = 10). Dimensionality reduction was performed by PCA followed by UMAP, and OPCs were identified by *Pdgfra* and *Cspg4* expression and extracted for subclustering. The OPC subset was re‐clustered using the Leiden algorithm (resolution = 0.4), yielding four subpopulations (OPC1–OPC4). Cell proportions and expression of *P2rx7* and *Nlrp3* were quantified and visualized via dot plots.

### 2.4 Animal Models and Experimental Cohorts

Specific pathogen‐free (SPF) Sprague–Dawley (SD) rat pups at postnatal Day 3 (P3) were used. The brain developmental stage of P3–P5 neonatal rats closely parallels that of human preterm/term infants, providing a suitable model of perinatal inflammatory brain injury. Perinatal white matter injury was induced by intracerebroventricular (i.c.v.) injection of lipopolysaccharide (LPS, 1 mg/kg; Sigma‐Aldrich) as previously described [20, 21, 22]. Briefly, P3 pups were anesthetized and fixed on a neonatal stereotaxic apparatus. LPS was injected into the left lateral ventricle using a 2 μL microsyringe at the following coordinates relative to lambda: anteroposterior −1.1 mm, mediolateral −1.5 mm, dorsoventral −2.5 mm. The needle was left in place for 1 min to prevent reflux. Pups were randomly assigned to seven groups: Sham, *Costunolide* (Cos) alone, LPS, and LPS + Cos (1.25, 2.5, 5, and 10 mg/kg). Cos was administered intraperitoneally once daily for 5 consecutive days, starting 2 h before LPS injection. For mechanistic analyses at P4, the optimal dose of 5 mg/kg Cos was used. Sham and Cos‐alone groups received equal volumes of vehicle via both i.p. and i.c.v. routes. To validate the P2X7‐dependent mechanism, a global *P2rx7* knockout rat strain (SD‐*P2rx7* ^em1Smoc^) was generated by CRISPR/Cas9 (Shanghai Model Organisms Center Inc.). Deletion of exons 2–11 introduced a frameshift and premature termination, ensuring functional P2X7 ablation. Genetic cohorts included WT Sham, WT + LPS, *P2rx7* ^−/−^, and *P2rx7* ^−/−^ + LPS groups.

### 2.5 Primary Oligodendrocyte Precursor Cell (OPC) Isolation and Culture

Primary OPCs were isolated from the cerebral cortices of neonatal rats at P1–P2 following our well‐established protocols [23, 24, 25]. Prior to targeted interventions, the purity and stage‐specific phenotype of the primary cultures were rigorously authenticated using canonical OPC surface markers (A2B5 and PDGFRα), with purity ≥ 93%. The isolated cells exhibited the characteristic bipolar or tripolar morphologies (Figure 2A,B), and only highly purified cultures were utilized for subsequent experiments.

### 2.6 In Vitro Model Construction and Targeted Interventions

To establish the in vitro inflammatory injury model, purified primary OPCs were stimulated with LPS (1 μg/mL) for 24 h. For pharmacological interventions, OPCs were pre‐treated with Cos or the selective P2X7 antagonist A‐438079 (#S7705, Selleck Chemicals, Houston, TX, USA) at their optimal concentrations. To confirm P2X7 specificity, gene silencing was performed using a *P2rx7*‐targeted siRNA or a non‐targeting control (siRNA‐NC) with a non‐lipid cationic polymer transfection reagent. Experimental groups were: Sham, Cos, LPS, LPS + Cos, LPS + A‐438079, LPS + siRNA‐*P2rx7*, and LPS + siRNA‐NC.

### 2.7 Immunofluorescence Staining, Imaging, and Computational Analysis

Cells were incubated overnight at 4°C with the following primary antibodies: mouse anti‐A2B5 (1:100; Cat# ab53521, Abcam), mouse anti‐PDGFRα (1:20; Cat# sc‐398,206, Santa Cruz Biotechnology), rabbit anti‐PDGFRα (1:100; Cat# AF0241, Affinity Biosciences), rabbit anti‐P2X7 (1:100; Cat# ER1901‐99/ER1901‐50, HUABIO), mouse anti‐P2X7 (1:100; Cat# BF8403, Affinity Biosciences), mouse anti‐NLRP3 (1:100; Cat# BF8029, Affinity Biosciences), and rabbit antibodies against Cleaved‐Caspase 1 (Asp296) (1:100; Cat# AF4005, Affinity Biosciences), Gasdermin D (N‐terminal) (1:100; Cat# HA721144, HUABIO), Cleaved‐IL‐1β (Asp116) (1:100; Cat# AF4006, Affinity Biosciences), rat anti‐MBP (1:20, Abcam, RRID: AB_305869, Cambridge, UK), and rabbit anti‐CNPase (1:100, Cat# AF4084, Affinity Biosciences, Jiangsu, China). After washing, cells were incubated for 1 h at room temperature with FITC‐ or Cy3‐conjugated secondary antibodies (Proteintech: Cat# SA00003‐1, SA00009‐1, SA00003‐2, SA00009‐2) matched to the primary species. Nuclei were counterstained with DAPI. Morphological evaluation of pyroptotic effectors (Cleaved Caspase‐1, GSDMD, and Cleaved IL‐1β) was performed using a standard fluorescence microscope. Subcellular colocalization and expression profiling (e.g., PDGFRα/P2X7 and P2X7/NLRP3) were captured using a confocal laser scanning microscope.

### 2.8 Combinational Stress Matrix and Cell Viability Assay

To comprehensively map the protective efficacy of Cos against P2X7‐dependent pyroptosis, a two‐dimensional cell survival matrix was constructed. OPCs seeded in 96‐well plates were subjected to a combinational treatment matrix comprising a concentration gradient of Cos alongside escalating doses of exogenous ATP. Cell viability was dynamically quantified using CCK‐8, with optical density measured at 450 nm.

### 2.9 Intracellular Calcium Imaging

Intracellular calcium dynamics were monitored using the fluorescent calcium indicator Fluo‐4 AM (Cat# S1060, Beyotime Biotechnology, Shanghai, China). Dynamic fluorescence signals representing intracellular calcium transients (Figure 2Q) were captured. The relative mean fluorescence intensity of Fluo‐4 across experimental cohorts was quantified using ImageJ software.

### 2.10 Endogenous co‐Immunoprecipitation (Co‐IP) Assay

To validate the endogenous P2X7–NLRP3 interaction, reciprocal Co‐IP was performed in OPCs primed with LPS (1 μg/mL, 23 h) and ATP (3 mM, 1 h) to induce inflammasome assembly. Cells were lysed on ice in immunoprecipitation lysis buffer containing protease inhibitors. After centrifugation (12,000 × g, 15 min, 4°C) and BCA quantification, lysates were divided into Input, IgG isotype control, and IP groups. For forward Co‐IP, proteins were incubated overnight at 4°C with anti‐P2X7 antibody or rabbit IgG control; for reverse Co‐IP, anti‐NLRP3 antibody or mouse IgG1 control was used, with an aliquot of original lysate reserved as Input. Protein A/G magnetic beads were added and incubated for 1–2 h at 4°C with rotation. Immunocomplexes were washed, eluted, resolved by SDS‐PAGE, and transferred to PVDF membranes for sequential probing of target and bait proteins.

### 2.11 Western Blot Analysis

Protein lysates were prepared from OPCs and brain tissues, quantified by BCA assay, separated by SDS‐PAGE, and transferred to PVDF membranes. After blocking, membranes were incubated overnight at 4°C with primary antibodies: mouse monoclonal anti‐NLRP3 (1:100, Cat# BF8029, Affinity Biosciences), rabbit polyclonal anti‐Cleaved Caspase‐1 (Asp296) (1:100, Cat# AF4005, Affinity Biosciences), rabbit monoclonal anti‐Gasdermin D N‐terminal fragment (GSDMD‐NT) (1:100, Cat# HA721144, HUABIO), rabbit polyclonal anti‐IL‐18 (1:100, Cat# DF6252, Affinity Biosciences), and rabbit polyclonal anti‐Cleaved IL‐1β (Asp116) (1:100, Cat# AF4006, Affinity Biosciences). Owing to their well‐separated molecular weights, NLRP3 and IL‐18 were probed simultaneously on the same membrane. Immunoreactive bands were detected with HRP‐conjugated secondary antibodies and ECL. Membranes were stripped and re‐probed for β‐actin as loading control. Band intensities were quantified with ImageJ and normalized to β‐actin.

### 2.12 Statistical Analysis

Data normality and variance homogeneity were assessed using the Shapiro–Wilk test and Brown–Forsythe test, respectively. For normally distributed data with equal variances, two‐group comparisons used an unpaired two‐tailed Student's *t*‐test, and multi‐group comparisons used one‐way ANOVA followed by Tukey's post hoc test. For normally distributed data with unequal variances, two‐group comparisons used Welch's *t*‐test, and multi‐group comparisons used Welch's one‐way ANOVA followed by Games–Howell post hoc test. For non‐normally distributed data, non‐parametric tests were applied: Mann–Whitney *U* test for two groups, and Kruskal–Wallis test with Dunn's post hoc test for multiple groups. Sample sizes were determined based on established neonatal neuroinflammation models; outlier testing was not performed. Data are presented as the mean ± standard deviation (SD) or as box‐and‐whisker plots (center line, median; box limits, first and third quartiles; whiskers, minimum to maximum). Statistical analyses were conducted using SPSS 25.0 (RRID: SCR_002865; IBM), and graphs were generated with Prism 8.02 (RRID: SCR_002798; GraphPad). Statistical significance was defined as *p* < 0.05.

## 3 Results

### 3.1 Species‐Specific Binding Characteristics and Stability of P2X7‐*Costunolide* Complexes: In Silico Simulations and in Vitro Validation

Molecular docking, 100 ns MD simulations, and MMPBSA analysis showed that Cos binds to the extracellular ATP‐binding domain of both human and rat P2X7 receptors, physically occluding the channel entrance/exit pathway (Figure 1A–H and Videos S1–S4). Both complexes remained stable, with RMSD fluctuations of 0.0–0.8 nm (human) and 0.0–1.2 nm (rat) (Figure 1B–I). The human complex was more compact (Rg 4.5–5.0 nm) than the rat complex (4.5–5.5 nm) (Figure 1C–J). SASA and hydrogen bond analyses indicated stable hydrophobic interfaces and stronger H‐bond stabilization in the rat complex (Figure 1D,E,K,L). MMPBSA revealed stronger binding for the rat complex (ΔTOTAL = −18.39 ± 1.74 kcal/mol) than the human complex (−11.67 ± 2.15 kcal/mol), driven mainly by van der Waals forces (Figure 1G–N), and FEL confirmed superior thermal stability of the rat complex (Figure 1O–R). SPR supported moderate‐affinity binding of Cos to human P2X7 (*K*_a_ = 1.33 × 10^5^ M^−1^ s^−1^, *K*_d_ = 4.08 × 10^−1^ s^−1^, and *K*_D_ = 3.08 μM) (Figure 1S).

**FIGURE 1:**
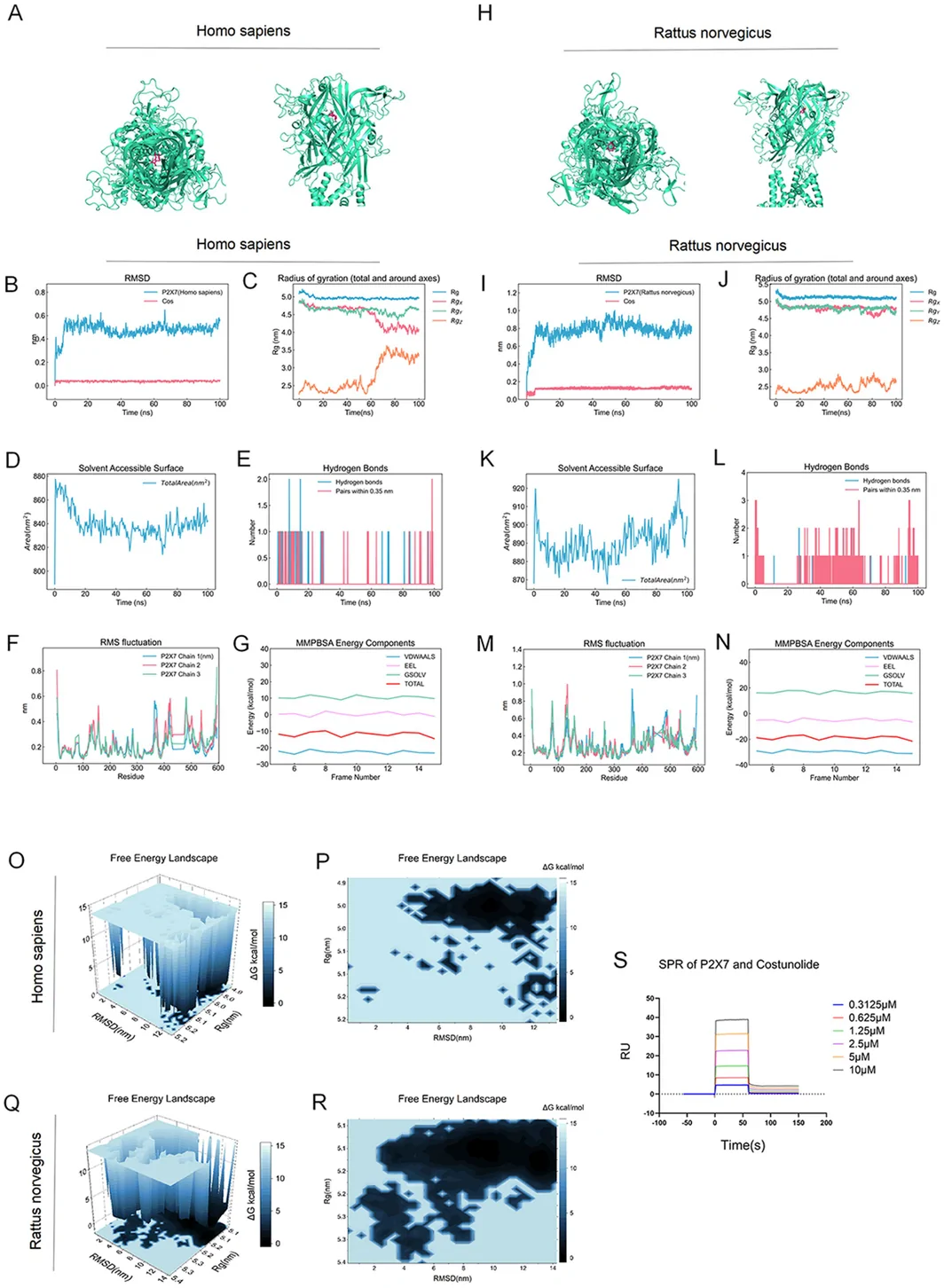
Structural, Energetic, and Kinetic Characterization of *Costunolide* (Cos) Binding to Human and Rat P2X7 Receptors. (A, H) Representative 3D binding modes of the P2X7‐Cos complex. (A) *Homo sapiens* (Human) complex (longitudinal view) and (H) *Rattus norvegicus* (Rat) complex (transverse view), magnified at the extracellular ATP‐binding domain. (B, I) Time evolution of the Root Mean Square Deviation (RMSD) for the protein backbone over 100 ns MD simulations for (B) Human and (I) Rat complexes. (C, J) Structural compactness and stability analysis. (C) Structural characterization of the Human P2X7‐Cos complex; (J) Radius of Gyration (Rg) profile for the Rat P2X7‐Cos complex, showing total Rg and axial components (Rg_x_, Rg_y_, Rg_z_). (D, K) Solvent Accessible Surface Area (SASA) fluctuations calculated over the 100 ns trajectory for (D) Human and (K) Rat complexes. (E, L) Analysis of hydrogen bond interactions (donor‐acceptor distance ≤ 0.35 nm) between P2X7 and Cos for (E) Human and (L) Rat complexes. (F, M) Residue‐level flexibility assessed by Root Mean Square Fluctuation (RMSF) of Chains 1–3 for (F) Human and (M) Rat receptors.(G, N) Binding free energy decomposition. Energy components (van der Waals [ΔVDWAALS], electrostatic [ΔEEL], solvation [ΔGSOLV], and total binding energy [ΔTOTAL]) calculated via MMPBSA analysis for (G) Human and (N) Rat complexes. (O, R) Free Energy Landscapes (FEL). (O) 3D ΔG distribution and (P) 2D structural‐energetic projection for the Human complex; (Q) 3D FEL and (R) 2D FEL for the Rat complex. (S) Surface Plasmon Resonance (SPR) sensorgram validating the interaction between Human P2X7 protein and Cos. The plot displays reference‐subtracted Response Units (RU) versus time (s) across a concentration gradient (0.3125, 0.625, 1.25, 2.5, 5, and 10 μM).

### 3.2 *Costunolide* Attenuates NLRP3 Inflammasome Activation in OPCs via Modulation of P2X7 Channel Gating and Physical Interaction

OPCs were validated by A2B5 immunoreactivity (Figure 2A). Baseline P2X7 expression on PDGFRα^+^ OPCs remained unchanged across Sham, Cos, LPS, and LPS + Cos groups (Figure 2B,C), whereas LPS markedly upregulated NLRP3, which co‐localized with P2X7 at the cell periphery (Figure 2D). Cos significantly suppressed this LPS‐induced NLRP3 upregulation and membrane recruitment, phenocopying the effects of the P2X7 antagonist A‐438079 and siRNA‐*P2rx7* knockdown (Figure 2D,E). Fluorescence intensity profiles confirmed synchronized attenuation of P2X7 and NLRP3 signals at the plasma membrane (Figure 2F–L). Reciprocal Co‐IP suggested an endogenous P2X7–NLRP3 interaction, supporting a receptor‐anchored inflammasome assembly model (Figure 2M,N). Faint bands in the IgG control likely reflect non‐specific background; specific bands in the IP groups were markedly stronger, and reciprocal Co‐IP confirmed the interaction. Functionally, Cos conferred dose‐dependent protection, with ~10 μM identified as the optimal concentration across LPS and ATP stress matrices (Figure 2O,P). Fluo‐4 AM imaging further showed that Cos significantly reduced LPS‐ or ATP‐induced intracellular calcium overload (Figure 2Q,R). Collectively, Cos may act through a dual mechanism: modulating P2X7–NLRP3 complex assembly and restricting P2X7‐mediated calcium influx.

**FIGURE 2:**
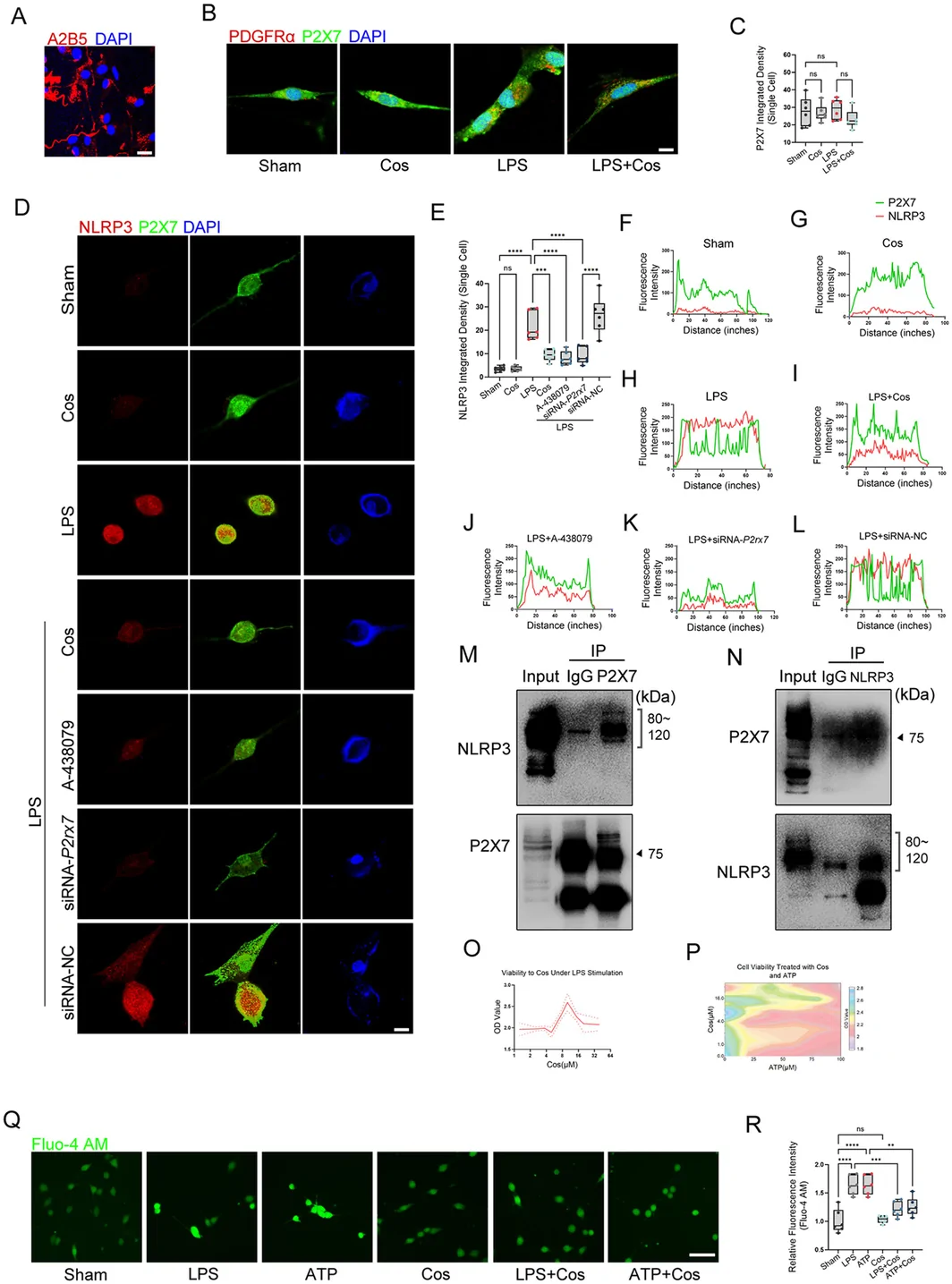
*Costunolide* (Cos) restricts NLRP3 inflammasome activation through the modulation of P2X7 channel gating and physical interaction. (A) Immunofluorescence morphology of primary OPCs identified by A2B5 (red) and DAPI (blue). (B, C) Confocal representative images (B) and single‐cell integrated density quantification (C) of PDGFRα (red) and P2X7 (green) across Sham, Cos, LPS, and LPS + Cos cohorts. (D, E) Representative confocal immunofluorescence images (D) and corresponding quantification (E) evaluating the spatial co‐localization of NLRP3 (red) and P2X7 (green) at the cell periphery in OPCs subjected to Sham, Cos, LPS, and LPS co‐treated with Cos, A‐438079, siRNA‐P2X7, or siRNA‐NC. (F–L) Fluorescence intensity spatial plot profiles of P2X7 (green) and NLRP3 (red) across the indicated experimental groups, mapping signal synchronization predominantly at the plasma membrane boundary. (M, N) Reciprocal co‐immunoprecipitation (Co‐IP) assays characterizing the endogenous physical interaction between P2X7 and NLRP3 proteins in OPCs. (O, P) CCK‐8 cell viability assessments of OPCs exposed to varying Cos concentration gradients under LPS stimulation (O) and combinational stress mapping with Cos and exogenous ATP (P).(Q, R) Representative Fluo‐4 AM fluorescence imaging (Q) and relative mean fluorescence intensity (MFI) quantification (R) of intracellular calcium (Ca^2+^) levels in OPCs across Sham, LPS, ATP, Cos, LPS + Cos, and ATP + Cos groups. Data are presented as box‐and‐whisker plots or mean ± SD from at least six independent experiments (*n* ≥ 6). **p < 0.01, ***p < 0.001, ****p < 0.0001; ns, not significant. Scale bars: 100 μm in (A) and (Q); 20 μm in (B) and (D).

### 3.3 *Costunolide* Abrogates LPS‐Induced NLRP3‐Dependent Pyroptosis and Pro‐Inflammatory Cytokine Maturation in OPCs

Immunofluorescence revealed that LPS markedly upregulated Cleaved Caspase‐1 (Figure 3A–D) and Cleaved IL‐1β (Figure 3C–F), and induced GSDMD redistribution into ring‐like puncta at the plasma membrane (Figure 3B–E) specifically in P2X7^+^ OPCs. Cos treatment restricted GSDMD to the cytoplasm and suppressed Caspase‐1/IL‐1β activation to Sham levels (Figure 3A–F). Immunoblotting confirmed that LPS increased NLRP3 expression (Figure 3G,H), promoted Caspase‐1 p20 and GSDMD‐NT generation (Figure 3I–L), and elevated IL‐18 and cleaved IL‐1β (Figure 3M–Q). Cos broadly suppressed this NLRP3/Caspase‐1/GSDMD axis, inhibiting both protein upregulation and proteolytic cleavage.

**FIGURE 3:**
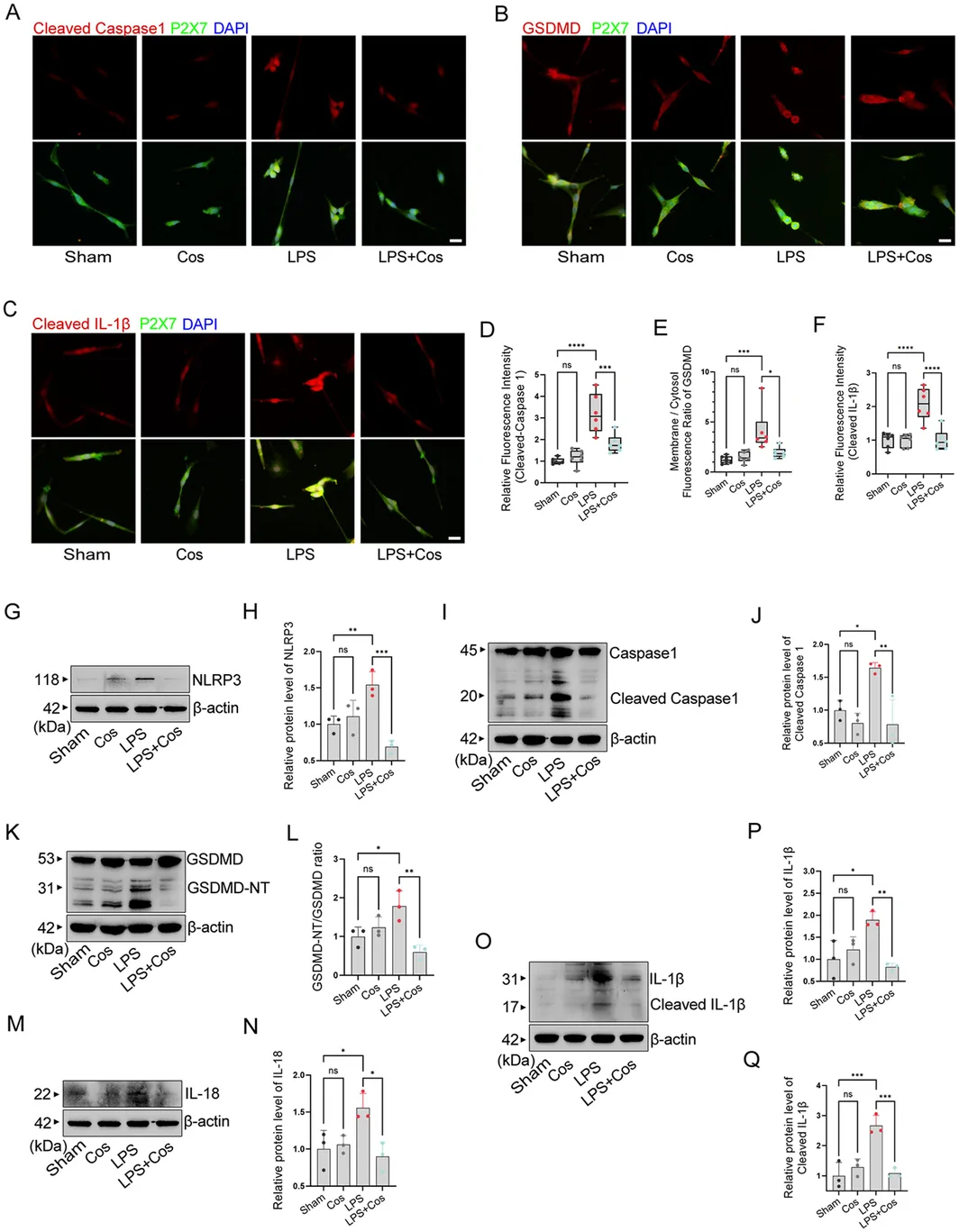
*Costunolide* (Cos) abrogates LPS‐induced NLRP3‐dependent pyroptosis and pro‐inflammatory cytokine maturation in OPCs. (A–C) Representative confocal images detailing the subcellular expression of P2X7 alongside Cleaved Caspase‐1 (A), GSDMD (B), and Cleaved IL‐1β (C). Nuclei were counterstained with DAPI (blue). (D–F) Quantification of relative fluorescence intensities for Cleaved Caspase‐1 (D) and Cleaved IL‐1β (F), and the membrane‐to‐cytosol fluorescence ratio mapping GSDMD translocation (E) across Sham, Cos, LPS, and LPS + Cos cohorts. (G, I, K, M, O) Representative immunoblots of NLRP3 (G), Caspase‐1 and Cleaved Caspase‐1 (I), full‐length GSDMD and GSDMD‐NT (K), IL‐18 (M), and IL‐1β and Cleaved IL‐1β (O) across the indicated treatments, with β‐actin serving as the loading control. (H, J, L, N, P, Q) Corresponding densitometric quantification for relative protein levels of NLRP3 (H), Cleaved Caspase‐1 (J), GSDMD‐NT to full‐length GSDMD ratio (L), IL‐18 (N), IL‐1β (P), and Cleaved IL‐1β (Q). Data are presented as box‐and‐whisker plots or mean ± SD. For single‐cell fluorescence quantification (D–F), *n* ≥ 30 cells per group from six independent cultures. For immunoblotting analyses, data represent at least three independent biological replicates (*n* ≥ 3). *p < 0.05, **p < 0.01, ***p < 0.001, ****p < 0.0001; ns, not significant. Scale bars: 50 μm.

### 3.4 *Costunolide* Curtails NLRP3‐Dependent Pyroptosis Strictly via the P2X7 Receptor Pathway

To determine whether Cos acts specifically through P2X7, its effects were compared with those of the P2X7 antagonist A‐438079 and siRNA‐mediated *P2rx7* knockdown. Under LPS stress, Cos suppressed Cleaved Caspase‐1 and Cleaved IL‐1β upregulation and restricted GSDMD membrane translocation to a degree indistinguishable from A‐438079 and siRNA‐*P2rx7* (Figure 4A–F). Immunoblotting confirmed that Cos, A‐438079, and *P2rx7* silencing similarly abrogated LPS‐induced NLRP3 overexpression, Caspase‐1 p20 and GSDMD‐NT generation, and IL‐1β/IL‐18 maturation (Figure 4G–Q). These congruent results indicate that Cos suppresses NLRP3‐dependent pyroptotic signaling specifically by antagonizing P2X7.

**FIGURE 4:**
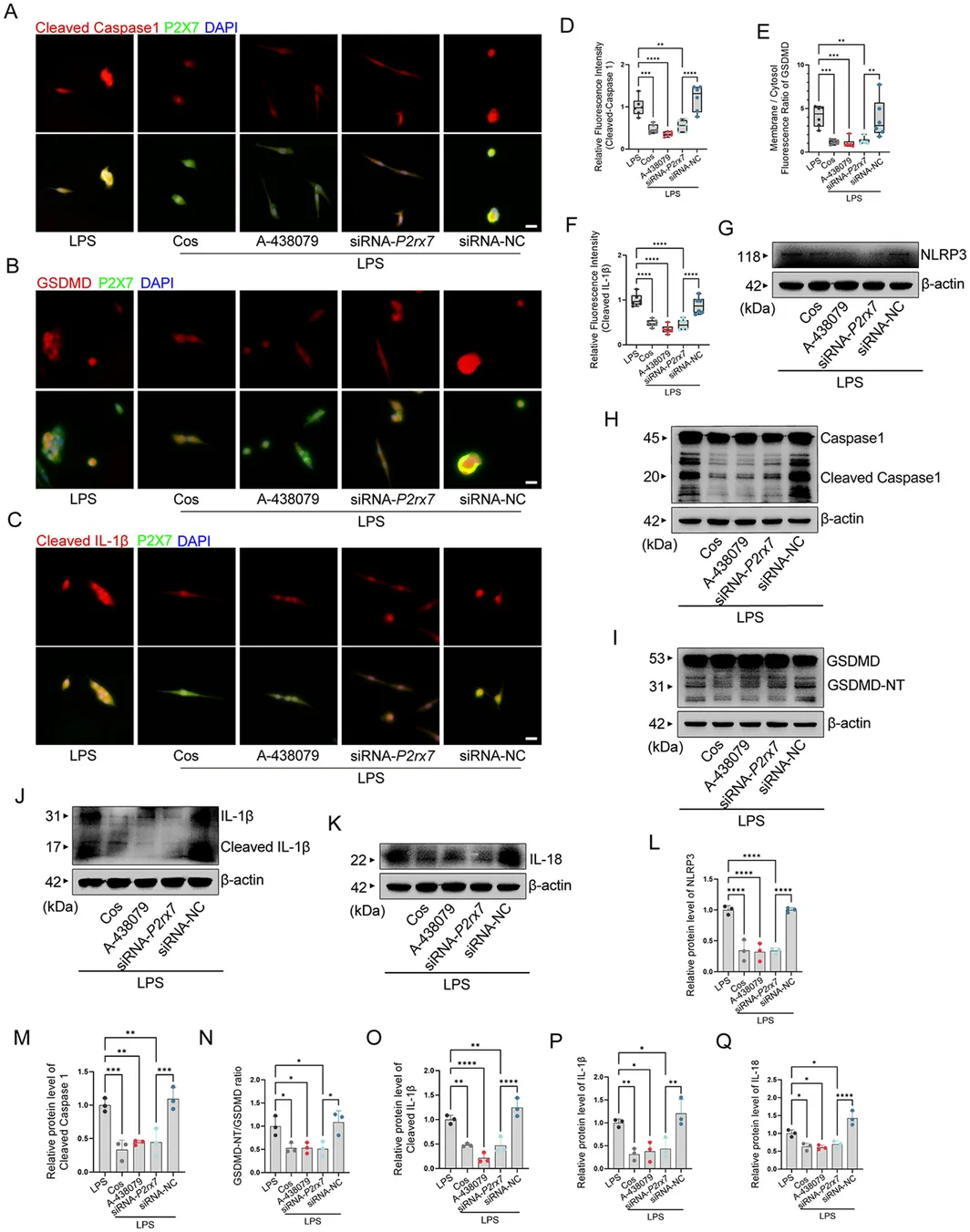
*Costunolide* curtails NLRP3‐dependent pyroptosis strictly via the P2X7 receptor pathway. (A–C) Representative confocal immunofluorescence images detailing the subcellular expression of P2X7 alongside Cleaved Caspase‐1 (A), GSDMD (B), and Cleaved IL‐1β (C). Nuclei were counterstained with DAPI (blue). Experimental groups include LPS exposure alone and LPS co‐administered with Cos, A‐438079, siRNA‐*P2rx7*, or siRNA‐NC. (D–F) Quantification of relative fluorescence intensities for Cleaved Caspase‐1 (D) and Cleaved IL‐1β (F), and the membrane‐to‐cytosol fluorescence ratio mapping GSDMD translocation (E) across the indicated cohorts. (G–K) Representative immunoblots of NLRP3 (G), Caspase‐1 and Cleaved Caspase‐1 (H), full‐length GSDMD and GSDMD‐NT (I), IL‐1β and Cleaved IL‐1β (J), and IL‐18 (K) across the specified treatments, with β‐actin serving as the internal loading control. (L–Q) Corresponding densitometric quantification for relative protein levels of NLRP3 (L), Cleaved Caspase‐1 (M), GSDMD‐NT to full‐length GSDMD ratio (N), Cleaved IL‐1β (O), IL‐1β (P), and IL‐18 (Q). Data are presented as box‐and‐whisker plots or mean ± SD. For single‐cell fluorescence quantification (D–F), *n* ≥ 30 cells per group from six independent cultures. For immunoblotting analyses (L–Q), data represent at least three independent biological replicates (*n* ≥ 3). *p < 0.05, **p < 0.01, ***p < 0.001, ****p < 0.0001. Scale bars: 50 μm.

### 3.5 Single‐Cell Transcriptomic Analysis Reveals an Injury‐Emergent OPC Subpopulation With Elevated *P2rx7* and *Nlrp3* Co‐Expression

To characterize OPC transcriptional heterogeneity under neuroinflammatory conditions, we analyzed a public scRNA‐seq dataset (GSE225580) from neonatal mouse brains subjected to Ctrl or hypoxia‐ischemia (HI)‐induced WMI. OPCs were identified by canonical markers *Pdgfra* and *Cspg4* and re‐clustered at resolution 0.4, revealing four subpopulations (OPC1–OPC4) (Figure 5E,F). Cell proportion analysis revealed marked injury‐induced remodeling: the homeostatic OPC1 subset declined from 84.5% in Ctrl to 2.4% after WMI, and OPC4 from 9.9% to 1.5%, whereas OPC2 expanded to 91.2% of OPCs. Transcriptomic profiling showed that OPC2 retained *Pdgfra/Cspg4* expression and contained 16% *P2rx7* ^+^ and 1.8% *Nlrp3* ^+^ cells; its numerical dominance generated a substantial P2X7‐expressing pool in injured white matter. The smaller OPC3 subset exhibited the highest *P2rx7* positivity (27.6%) and was the primary *Nlrp3*‐expressing niche (3.4%), whereas *Nlrp3* was absent in OPC1. Collectively, these findings reveal a shift from homeostatic OPC1/OPC4 states to an injury‐responsive, *P2rx7–Nlrp3‐primed* OPC repertoire dominated by OPC2 and OPC3.

**FIGURE 5:**
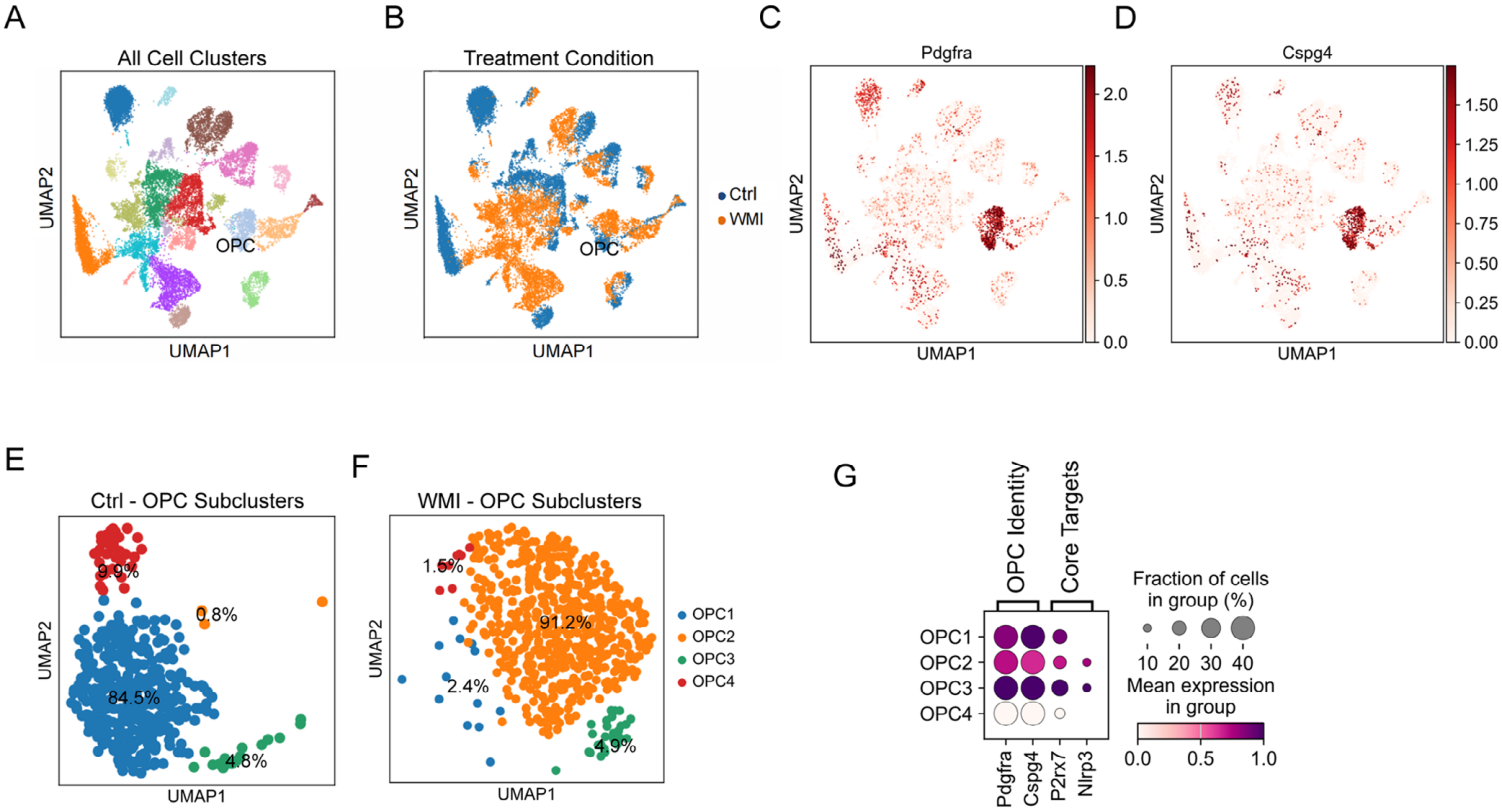
Single‐cell transcriptomic profiling reveals a “pyroptosis‐prone” OPC subpopulation following inflammatory insult. (A, B) UMAP visualizations of global cell clusters from neonatal mouse brains, color‐coded by cell identity (A) and treatment condition (Ctrl vs. WMI) (B). (C, D) Feature plots showing the distribution of canonical OPC markers *Pdgfra* (C) and *Cspg4* (D) used for population isolation. (E, F) High‐resolution UMAP embeddings of the re‐clustered OPC population in Ctrl (E) and WMI (F) groups. Note the massive expansion of the OPC2 subset (91.2%) and depletion of the homeostatic OPC1 subset (2.4%) in the injured cohort. (G) Dot plot displaying the expression of pan‐OPC identity genes (*Pdgfra*, *Cspg4*) and core pyroptosis‐related targets (*P2rx7*, *Nlrp3*). In the Ctrl group, OPC1 is the dominant subset and *Nlrp3* is undetectable; following WMI, OPC1 is largely replaced by OPC2 (91.2%), while a small OPC3 population (27.6% *P2rx7* ^+^, 3.4% *Nlrp3* ^+^) persists as the most inflammasome‐primed subset. A total of 917 OPCs (375 Ctrl, 542 WMI) were analyzed.

### 3.6 *Costunolide* Suppresses P2X7‐NLRP3 Inflammasome Activation and OPC Pyroptosis in Vivo

To validate the in vivo relevance of our in vitro findings, we assessed Cos efficacy in the developing periventricular white matter of LPS‐challenged rats at P4. Using a custom ImageJ segmentation plugin for single‐cell spatial resolution, we observed that P2X7^+^PDGFRα^+^ OPCs in the LPS group exhibited hypertrophic, irregular morphologies and broader spatial distribution compared with the small, bipolar OPCs in Sham controls, while baseline P2X7 positivity remained stable across groups (Figure 6A–C). LPS induced marked NLRP3 upregulation within OPCs, which was profoundly suppressed by Cos co‐administration (Figure 6B–D). Western blotting of brain tissues confirmed that Cos significantly attenuated LPS‐induced NLRP3 overexpression, Caspase‐1 p20 cleavage, GSDMD‐NT formation, IL‐18, and Cleaved IL‐1β upregulation (Figure 6E–O). These in vivo data demonstrate that Cos suppresses the P2X7–NLRP3 pyroptotic axis in the injured white matter.

**FIGURE 6:**
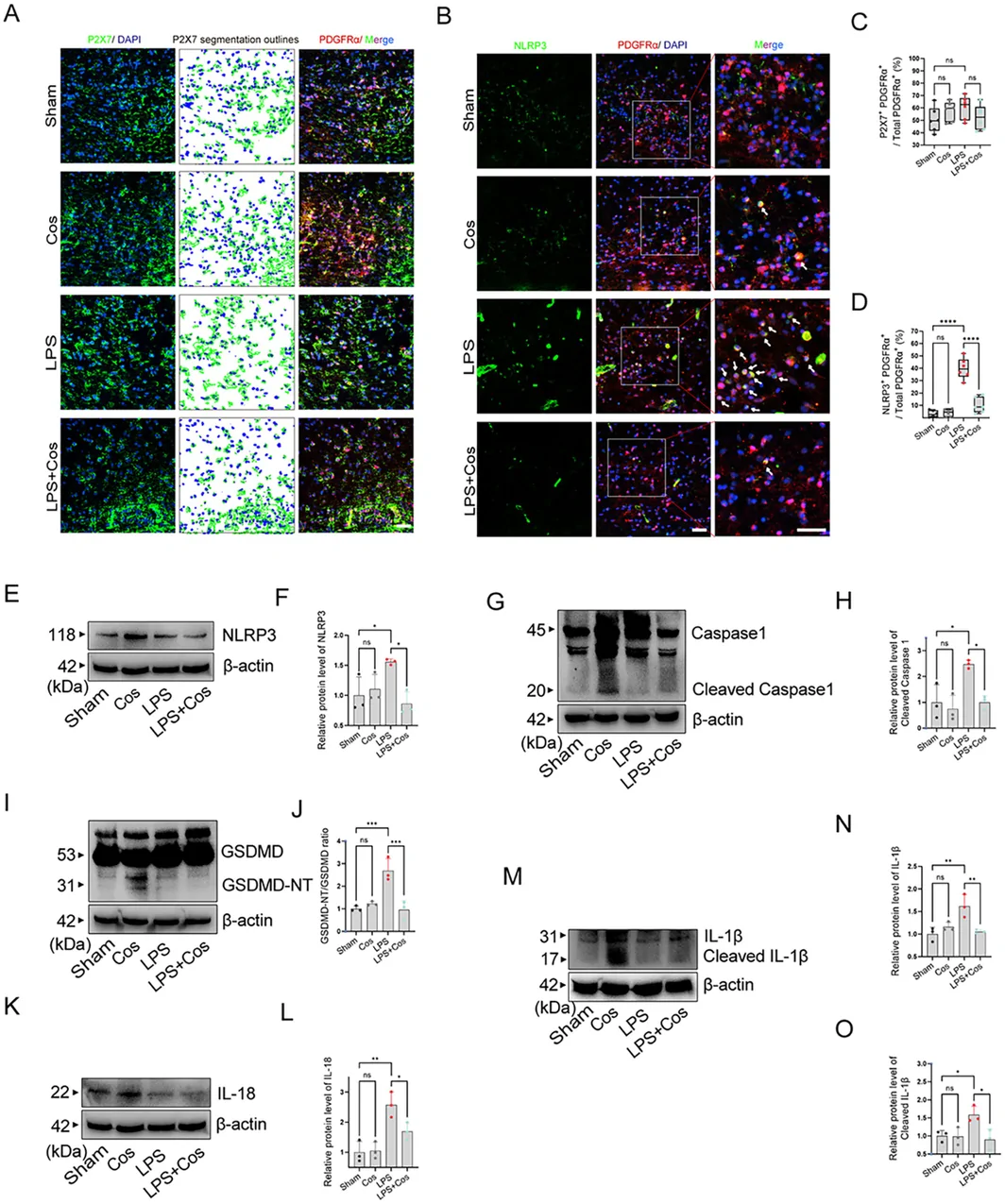
*Costunolide* suppresses LPS‐induced P2X7–NLRP3 inflammasome activation and OPC pyroptosis in vivo. (A, B) Representative images detailing the subcellular expression of P2X7 (A) and NLRP3 (B) (green) within PDGFRα^+^ OPCs (red) at P4. The segmentation outlines in (A) visually highlight the hypertrophic and highly irregular morphological signatures of P2X7^+^ cells following LPS insult, contrasting with their homeostatic counterparts. White arrows indicate the robust co‐localization of NLRP3 within these expanded OPC boundaries. (C, D) Stringent quantitative analysis utilizing the custom cell segmentation masks to determine the exact percentages of P2X7^+^PDGFRα^+^ cells (C) and NLRP3^+^PDGFRα^+^ cells (D) relative to the total PDGFRα^+^ OPC pool. (E, G, I, K, M) Representative immunoblots of NLRP3 (E), Caspase‐1 and Cleaved Caspase‐1 (G), full‐length GSDMD and GSDMD‐NT (I), IL‐18 (K), and IL‐1β and Cleaved IL‐1β (M) extracted from brain tissues across Sham, Cos, LPS, and LPS + Cos groups, with β‐actin serving as the loading control. (F, H, J, L, N, O) Corresponding densitometric quantification for the relative protein levels of NLRP3 (F), Cleaved Caspase‐1 (H), GSDMD‐NT to full‐length GSDMD ratio (J), IL‐18 (L), IL‐1β (N), and Cleaved IL‐1β (O). Data are presented as box‐and‐whisker plots or mean ± SD. Data represent independent biological replicates (*n* ≥ 3 animals per group). *p < 0.05, **p < 0.01, ***p < 0.001, ****p < 0.0001; ns, not significant. Scale bars: 100 μm.

### 3.7 *Costunolide* Rescues Myelin Architecture and Promotes Oligodendrocyte Maturation Following Inflammatory Insult

At P7, LPS severely disrupted myelin integrity, reducing MBP and CNPase fluorescence and producing fragmented myelin tracts compared with Sham controls (Figure S1A–C). Cos co‐administration dose‐dependently restored these deficits, with 5 mg/kg significantly improving MBP^+^ fiber architecture and MBP–CNPase colocalization, indicating proper assembly of compact and non‐compact myelin. This protection persisted through P14 (Figure S1D): Cos (notably at 2.5, 5, and 10 mg/kg) durably upregulated MBP (Figure S1E), whereas CNPase recovery exhibited an optimal window at 5 mg/kg (Figure S1F). Cos alone did not affect baseline myelination in healthy controls, confirming its specific protective action against inflammatory demyelination without intrinsic toxicity.

### 3.8 *P2rx7* Deficiency Confers Sustained Protection Against Inflammatory Myelin Deficits From the Early to Mature Developmental Stages

Immunofluorescence analysis at P7 showed that LPS challenge in WT rats reduced MBP and CNPase signals, whereas *P2rx7* deficiency substantially mitigated these deficits, with *P2rx7* ^−/−^ + LPS rats displaying preserved fasciculated myelin architecture (Figure S2A,C,D). This protection persisted through P14, as *P2rx7* ^−/−^ + LPS animals maintained higher MBP and CNPase intensities and fiber density compared with WT + LPS controls, which exhibited persistent hypomyelination (Figure S2B,E,F). Baseline myelination was comparable between *P2rx7* ^−/−^ and WT sham groups, indicating that P2X7 is dispensable for physiological myelination but critically drives pathological demyelination during neonatal neuroinflammation.

### 3.9 *P2rx7* Deficiency Abrogates LPS‐Induced NLRP3 Inflammasome Activation and OPC Pyroptosis in Vivo

Using P2rx7^−/−^ rats and a computational segmentation plugin to delineate PDGFRα^+^ OPCs at P4, we confirmed loss of P2X7 expression in KO groups, whereas WT rats showed robust P2X7 (Figure S3A–F). LPS challenge in WT rats markedly expanded NLRP3^+^, Cleaved Caspase‐1^+^, GSDMD‐NT^+^, and Cleaved IL‐1β^+^ cells, but these pyroptotic signatures were substantially reduced by *P2rx7* deletion (Figure S3B–E,G–J). Western blotting confirmed that Caspase‐1 p20, GSDMD‐NT, IL‐18, and Cleaved IL‐1β were all suppressed in *P2rx7* ^−/−^ + LPS brains relative to LPS alone (Figure S3K–U). These data provide genetic evidence that P2X7 is a critical upstream regulator of LPS‐induced NLRP3 activation and OPC pyroptosis.

## 4 Discussion

This study suggests that physical coupling of P2X7 and NLRP3 may function as a pyroptotic switch in OPCs during perinatal WMI, and that this complex can be disrupted by pharmacological or genetic blockade. Our findings suggest that P2X7 may act not only as a passive ion channel but as a scaffold facilitating inflammasome activation in non‐classical immune cells [26]. Furthermore, MD simulations and SPR suggest that *Costunolide* may bind and potentially occlude both human and rat P2X7 channels, providing a precise pharmacological strategy.

Previous studies in macrophages and microglia have shown that P2X7 can directly interact with NLRP3, either through adaptor proteins such as Paxillin or via direct intracellular domain binding [27, 28]. However, whether this physical coupling operates in the highly vulnerable OPC lineage during developmental white matter injury has remained unclear. Here, reciprocal Co‐IP in primary OPCs suggested an endogenous P2X7–NLRP3 interaction, and confocal imaging demonstrated LPS‐induced spatial colocalization of these proteins at the cell periphery. Co‐IP cannot distinguish direct from adaptor‐mediated binding, and the ion‐channel blockade by Cos likely contributes to pyroptosis inhibition. SPR with human P2X7 supports direct binding rather than rat‐specific affinity; however, MD predicted stronger binding of Cos to rat P2X7 with similar binding poses, and P2X7 is highly conserved [14, 15, 29], supporting species‐conserved binding. Mechanistically, Cos not only restricts pathological P2X7‐mediated calcium influx but also modulates P2X7–NLRP3 complex assembly, thereby suppressing Caspase‐1 cleavage, GSDMD membrane translocation, and IL‐1β/IL‐18 maturation. These effects closely phenocopy P2X7 antagonism and genetic knockdown, highlighting a novel therapeutic strategy for developmental white matter injury. scRNA‐seq further identified an injury‐emergent *P2rx7–Nlrp3*‐primed OPC subpopulation, reinforcing the role of this axis in OPC‐intrinsic vulnerability. GSE225580 (HI) serves as a mechanistic reference, not a direct representation of our rat LPS model; both model perinatal WMI, but species and insult differences may yield distinct OPC responses.

Given the substantial brain volume advantage of neonatal rats over mice—a factor that critically minimizes systemic physical errors during perinatal white matter injury modeling (such as localized microinjections) and significantly enhances experimental reproducibility—this study specifically generated and utilized a stably inherited *P2rx7* knockout (*P2rx7* ^*−/*−^) rat model, which remains relatively scarce globally. Although a few recent pioneering studies have employed CRISPR/Cas9 technology to generate *P2rx7* knockout rats and validated their utility in models of polycystic kidney disease [30], sex differences in renal artery contractility [31], and autoimmune glomerulonephritis [32], our research pioneers the innovative application of this robust genetic model within the field of developmental neuropathology. Using the *P2rx7* knockout rat model, we obtained convergent in vivo evidence linking pyroptotic blockade to functional recovery. High‐resolution spatial segmentation confirmed that *P2rx7* deletion abolished P2X7 expression and prevented LPS‐induced NLRP3 upregulation, Caspase‐1 cleavage, and GSDMD‐NT formation in OPCs (Figure S3). Functionally, this genetic blockade translated into sustained myelination benefits: *P2rx7* ^−/−^ + LPS rats exhibited preserved myelin architecture and MBP/CNPase expression at both P7 and P14, in contrast to the persistent hypomyelination observed in WT + LPS controls (Figure S2). However, global *P2rx7* knockout cannot exclude glial or microenvironmental contributions. Cos may also modulate microglial P2X7–NLRP3 signaling, indirectly protecting OPCs; such glial–OPC crosstalk may contribute to the sustained myelination protection in vivo. Future OPC‐specific or microglia–OPC co‐culture studies are needed to dissect these contributions.

Therapeutically, Cos is a natural sesquiterpene lactone with known neuroprotective and anti‐inflammatory potential [11]. In macrophages, Cos inhibits IL‐1β expression by downregulating MAPK cascades and AP‐1 activity [33]. In neonatal brain injury, Cos suppresses neuroinflammation via PI3K/AKT and promotes mitophagy via PINK1/Parkin [12, 13]. Here, we reposition Cos from an intracellular cascade modulator to a modulator of the P2X7–NLRP3 complex. MD simulations and SPR suggest that Cos may bind the extracellular ATP‐binding domain of human and rat P2X7 with moderate affinity (*K*_D_ = 3.08 μM), potentially occluding the channel entrance/exit and inducing allosteric effects.

This modulation of P2X7–NLRP3 signaling produced marked neuroprotective efficacy in vivo. In neonatal rats at P4, Cos suppressed LPS‐induced NLRP3 upregulation, Caspase‐1 cleavage, GSDMD‐NT formation, and pro‐inflammatory cytokine maturation, while also reversing the hypertrophic, irregular morphology of P2X7^+^ OPCs—achieving dual molecular and morphological restoration. This blockade translated into sustained myelination rescue: Cos dose‐dependently restored myelin architecture at P7 and maintained MBP/CNPase expression and fiber alignment at near‐normal levels through P14, with optimal efficacy at 5 mg/kg. These findings support the translational potential of Cos as a modulator for perinatal WMI. However, a limitation is that Cos efficacy was primarily validated in rodent models; its brain penetration and activity in human cells require further evaluation. As CNS‐penetrant P2X7 inhibitors such as JNJ‐54175446 [34] and JNJ‐55308942 [35] advance clinically, modulating this signaling network represents a promising therapeutic strategy for perinatal brain injury.

In conclusion, as summarized in our proposed working model (Graphical Abstract, Created in BioRender. feng, Z. (2026) https://BioRender.com/3u6tjms), this study establishes that P2X7–NLRP3 complex‐mediated “receptor‐anchored inflammasome assembly” is a central driver of OPC pyroptosis and perinatal WMI. Using *P2rx7* knockout rats, we confirmed in vivo that this axis acts as a critical driver of OPC pyroptosis and WMI. We further show that Cos may act as a modulator by associating with the P2X7 extracellular domain and modulating this complex, thereby halting GSDMD‐mediated pyroptosis and rescuing myelination. This provides a rationale for developing structural modulators as neurotherapeutics. Future studies should explore P2X7‐mediated glial‐neuronal communication and receptor polymorphism‐related susceptibility.

## Author Contributions

F.Z., Q.W. and T.L. conceptualized and designed the study; validated, analyzed, and curated the data; performed the experiments; performed software‐related analysis; and wrote and revised the manuscript. X.L., M.S., L.L. and M.Q. performed the experiments and contributed to writing the original draft. S.M., J.S. and W.Y. validated and curated the data, and performed visualization. H.J. and Y.L. conceived and designed the experiments, acquired funding, obtained resources, supervised the study, performed administration of the project, and critically revised the manuscript. All authors read and approved the final manuscript.

## Funding

This work was financially supported by the 2022 Science and Technology Benefit People Special Project from the Science and Technology Bureau of Qingdao West Coast New Area (2022‐54), the Shandong Medical and Health Science and Technology Project (02406030908), the Scientific Research Special Funding from the Wu Jieping Medical Foundation (AAMA.26.1‐VK‐26‐K‐3), the Beijing Medical and Health Foundation, and the Hospital Management Research Project of the Shandong Health Human Resources Management Association (SDHRGL202514).

## Ethics Statement

All animal experimental procedures were approved by the Institutional Animal Care and Use Committee of the Affiliated Hospital of Qingdao University (Approval No.: QYFYWZLL29114) and were performed in compliance with the ARRIVE guidelines and the National Institutes of Health Guide for the Care and Use of Laboratory Animals. This study did not involve human participants, human data, or human tissue; consent to participate is therefore not applicable.

## Conflicts of Interest

The authors declare no conflicts of interest.

## Supporting information


**Figure S1:** cns71263‐sup‐0001‐FigureS1.tif. *Costunolide* rescues myelin architecture and promotes oligodendrocyte maturation following LPS exposure in a dose‐dependent manner (A–D) Representative confocal immunofluorescence images of mature oligodendrocyte markers MBP (green) and CNPase (red) in the corpus callosum at postnatal Day 7 (P7; A) and Day 14 (P14; D). Nuclei were counterstained with DAPI (blue). Experimental groups encompass Sham, Cos monotherapy (10 mg/kg), LPS, and LPS co‐administered with graded Cos concentrations (1.25, 2.5, 5, and 10 mg/kg). (B, C) Quantification of mean fluorescence intensities for MBP (B) and CNPase (C) at P7. (E, F) Quantification of mean fluorescence intensities for MBP (E) and CNPase (F) at P14. Data are presented as box‐and‐whisker plots. Data represent independent biological replicates (*n* = 6 animals per group). *p < 0.05, **p < 0.01, ***p < 0.001, ****p < 0.0001; ns, not significant. Scale bars: 100 μm.

**Figure S2:** cns71263‐sup‐0002‐FigureS2.tif.*P2rx7* deficiency provides sustained restoration of myelin integrity and promotes oligodendrocyte maturation from P7 to P14 (A, B) Representative immunofluorescence images of mature oligodendrocyte markers MBP (green) and CNPase (red) in the periventricular white matter at postnatal Day 7 (P7; A) and Day 14 (P14; B). Nuclei were counterstained with DAPI (blue). Experimental groups include WT (Sham, LPS) and *P2rx7* ^−/−^(*P2rx7* ^−/−^, *P2rx7* ^−/−^ + LPS) cohorts. Note the sustained preservation of fasciculated myelin architecture in the *P2rx7* ^−/−^ + LPS group across both developmental stages. (C, D) Quantification of mean fluorescence intensities (MFI) for MBP (C) and CNPase (D) at P7. (E, F) Quantification of mean fluorescence intensities (MFI) for MBP (E) and CNPase (F) at P14. Data are presented as box‐and‐whisker plots. Data represent independent biological replicates (*n* = 6 animals per group). *p < 0.05, **p < 0.01, ***p < 0.001, ****p < 0.0001; ns, not significant. Scale bars: 100 μm.

**Figure S3:** cns71263‐sup‐0003‐FigureS3.tif. *P2rx7* deficiency completely abrogates LPS‐induced NLRP3 inflammasome activation and OPC pyroptosis in vivo (A–E) Representative images identifying the expression of P2X7 within PDGFRα^+^ OPCs (A), and the expression of NLRP3 (B), Cleaved Caspase‐1 (C), Cleaved IL‐1β (D), and GSDMD‐NT (E) co‐stained with P2X7 across Sham, *P2rx7* ^−/−^, LPS, and *P2rx7* ^−/−^ + LPS cohorts at P4. In (A), the second panel from the left displays the custom‐algorithmic segmentation outlines, demonstrating the precision of our single‐cell‐based quantification by stripping away extracellular background fluorescence. (F–J) Quantitative analysis of the percentages of specific marker‐positive cells relative to the total PDGFRα^+^ pool for P2X7^+^ (F), and relative to total DAPI^+^ cells for NLRP3^+^ (G), Cleaved Caspase‐1^+^ (H), Cleaved IL‐1β^+^ (I), and GSDMD‐NT^+^ (J). Data were derived strictly from the algorithmic segmentation masks to ensure unbiased, single‐cell resolution. (K, M, O, Q, S) Representative immunoblots of NLRP3 (K), Caspase‐1/Cleaved Caspase‐1 (M), GSDMD/GSDMD‐NT (O), IL‐18 (Q), and IL‐1β/Cleaved IL‐1β (S) from brain tissues. (L, N, P, R, T, U) Corresponding densitometric quantification for relative protein levels. Data are presented as box‐and‐whisker plots or mea*n* ± SD (*n* ≥ 3 animals per group). *p < 0.05, **p < 0.01, ***p < 0.001, ****p < 0.0001; ns, not significant. Scale bars: 100 μm for (A); 50 μm for (B–E).

**Video S1:** Molecular dynamics simulation of the human P2X7–*Costunolide* complex (100 ns trajectory). Longitudinal view.

**Video S2:** Molecular dynamics simulation of the human P2X7–*Costunolide* complex (100 ns trajectory). Transverse view.

**Video S3:** Molecular dynamics simulation of the rat P2X7–*Costunolide* complex (100 ns trajectory). Longitudinal view.

**Video S4:** Molecular dynamics simulation of the rat P2X7–*Costunolide* complex (100 ns trajectory). Transverse view.

## Data Availability

The datasets used and/or analyzed during the current study, including detailed methodological parameters, are available from the corresponding author upon reasonable request.
